# Experimental Studies of Strain and Stress Fields in a Granular Medium Under Active Pressure Using DIC and Elasto-Optic Methods

**DOI:** 10.3390/ma19010172

**Published:** 2026-01-03

**Authors:** Magdalena Pietrzak

**Affiliations:** Faculty of Civil Engineering, Environmental and Geodetic Sciences, Koszalin University of Technology, Śniadeckich 2, 75-453 Koszalin, Poland; magdalena.pietrzak@tu.koszalin.pl

**Keywords:** strain fields, active pressure, stresses, glass granules, DIC method, photoelasticity, granular media

## Abstract

This study presents a novel experimental methodology enabling the synchronous observation of strain and stress evolution in granular backfill subjected to active earth pressure. A physical model of plane deformation was used in which a rigid retaining wall was gradually moved away from the ground while simultaneously recording, at each step, both displacement-based images for digital image correlation (DIC) and photoelastic pictures of the force-chain rearrangements. The results show that active failure develops gradually through narrow shear bands, initiated near the wall base and propagating towards the ground surface. A consistent inverse relationship between shear-strain location and photoelastic stress concentration was identified: low-strain zones within the shear wedge in the shear and volumetric strain images correspond to strong force-chain development, whereas high-strain zones (strain localization) correspond to local stress release. These findings provide new experimental evidence regarding the micromechanics of active pressure and offer comparative data for calibrating DEM (discrete element method) models and interpreting the reduced active pressures reported in confined granular backfills.

## 1. Introduction

The problem of earth pressure on retaining walls is one of the fundamental problems of soil mechanics and has been studied since its inception [1]. Earth pressure is one of the fundamental forces considered in the design of quays, underground and hydraulic structures, bridge abutments, retaining walls, and other structures. Knowledge of the magnitude and distribution of earth pressure is essential for effective design. Despite over two hundred years of intensive research in this field, sufficient agreement between theoretical models of earth pressure and experimental results has still not been achieved. This is due to the specific nature of granular media and the complex nature of their deformation, accompanied by the ability to localise deformations, a characteristic recognised in the 1990s as fundamental to the behaviour of granular materials [2]. The dependence of the magnitude and distribution of pressure on the manner in which a retaining structure can move was noted by Terzaghi [3]. From then until the 1980s, research centres focused on studying the dependence of earth pressure on the type of retaining-wall movement. To define this dependence, numerous centres conducted studies on physical models of retaining structures [4,5,6,7]. These studies led to the conclusion that both the magnitude and distribution of pressure, as well as the point of application of its resultant, depend on the type of displacements to which the wall is subjected under various conditions. As a result, it was determined that the magnitude of the pressure depends primarily on whether the retaining structure moves away from the ground (active pressure) or towards the ground (passive pressure). However, its distribution also depends significantly on the wall’s rotation, the centre of which can be located at any point, depending on the structure’s operating mode. Taking this into account, Dubrow [8] (in Harr, 1966) proposed simplified analytical relations for non-hydrostatic pressure distributions across various modes of retaining-wall movement. A pervasive programme of experimental research involving the measurement of pressure distribution and the recording of backfill deformation using X-ray equipment was initiated at the University of Cambridge in the 1960s and continued until the 1980s [9,10,11,12,13,14]. Rigid walls rotating towards and away from the backfill for various centres of rotation, as well as flexible walls, were investigated. The entire study was catalogued and described in Leśniewska’s work [15]. These studies showed that backfill pressure depends not only on the type but also on the magnitude of wall displacement. They also demonstrated a strongly nonlinear pressure distribution across all rigid-wall movement patterns and a heterogeneous soil deformation field with extensive shear-band systems [15]. Consequently, Bransby and Milligan [12] concluded that without a precise understanding of the deformation field behind the retaining wall, it is impossible to determine the stresses acting on it, which are closely related to soil deformations. However, in the 1970s and 1980s, measurement tools that enabled such precise analysis did not exist.

At the beginning of the 21st century, significant progress was made in CCD (Charge-Coupled Device) technology, leading to the development of high-resolution digital and video cameras. At the same time, new image analysis techniques (full-field techniques) have been developed, such as DIC (digital image correlation). They apply not only to soil media but also to other granular materials, such as organic grains [16,17]. Thanks to DIC, it is now possible to track ground deformation in physical models and real structures with high precision. For example, Khosravi et al. [18] applied the DIC method in laboratory tests of a rigid retaining wall subjected to translational displacement, obtaining visualisation of the development of a shear band in the ground. In turn, Tung et al. [19] employed the DIC technique to monitor the full-field displacements of real retaining walls, thereby enabling non-contact tracking of the structure’s deformations under in situ conditions.

In recent years, significant progress has been made in applying DIC techniques to analyse deformation in granular media. Numerous studies have demonstrated that DIC enables high-resolution tracking of strain localisation, progressive damage, and arching phenomena in granular backfills [20,21,22]. These advances confirm the growing role of image-based measurements in geotechnical modelling, especially in low-strain conditions, where conventional sensing techniques often fail to capture early micromechanical processes [23].

This work was followed by studies that combined experimental and theoretical analyses. Khosravi et al. [24,25] presented a theoretical model of the active pressure for a rigid wall in translation mode, the results of which were consistent with experimental observations using DIC. Then, using the discrete element method, the micromechanics of backfill behaviour were reconstructed. Xu et al. [26] observed in an extensive series of geoPIV (Particle Image Velocimetry) experiments that reducing the backfill width-to-wall height ratio leads to a sequential increase in the number of such “reflected” slip surfaces, thereby reducing the effective active wedge. This results in a significant reduction in the magnitude of active pressure compared to classical theories—for very narrow backfill, the measured wall pressure is clearly lower and has a nonlinear distribution.

Recent studies have increasingly emphasised the influence of backfill confinement on the magnitude and distribution of active earth pressure. Chen et al. [27] and Wang et al. [28] demonstrated that narrow, non-cohesive backfills generate significantly lower active pressures than those of classical methods. Similar conclusions were reached by Xiong et al. [29], who demonstrated a nonlinear reduction in active pressure under translational wall motion. These findings support the hypothesis that geometric confinement causes progressive bulging, which has important implications for both analytical predictions and experimental modelling [30].

Chen et al. [31] developed an analytical solution for the active pressure in a trapped unbound medium, indicating that reducing the backfill width reduces both the total pressure and the active pressure coefficient relative to the semi-infinite state. Medium- and large-scale experimental studies conducted between 2020 and 2025 provided extensive evidence of this. Patel and Deb [32] performed a series of laboratory tests on a 60 cm rigid rotational wall (rotation around the base). They found that the distribution of active pressure during wall rotation is nonlinear—in the upper part of the wall, stresses below the Rankine stress occur (due to soil relaxation). In contrast, in the lower part, the pressure is higher, but the maximum is not at the very bottom. This was demonstrated even more clearly by Bashir and Basha [33], who conducted large-scale tests (1.2 m high wall) for various widths of narrow backfill (b/H ratio from 0.2 to 0.7). Their measurements showed that at the lowest b/H ratio, the active pressure was reduced by approximately 30–35%. Bashir’s research thus confirmed the strong vault effect in narrow backfills—the soil weight is partially absorbed by friction against the side walls, so the actual load on the retaining structure is lower than that assumed by standard models. In parallel, significant progress has been made in numerical modelling of the earth-pressure problem. Wörden and Achmus [34] conducted three-dimensional finite element simulations to analyse the active pressure on a rigid wall in sands as a function of the contact-friction coefficient and the geometric proportions of the system. Zhao et al. [35] simulated a narrow backfill adjacent to a slope using the FELA method (finite-element limit analysis). They identified up to three different forms of the active slip wedge for varying geometric proportions of the system. Xu et al. [26], who combined geoPIV observations with numerical calculations to demonstrate the gradual development of progressive failure—from local shear bands to the formation of a whole slip surface—depending on the wall displacement mode (translation vs. rotation) and backfill geometry. Such simulations provide a valuable complement to experimental studies, allowing for the analysis of the influence of individual parameters (such as soil properties, wall roughness, drainage, etc.) on pressure values and distributions. Recent years have brought significant progress in understanding the earth pressure on retaining walls. Thanks to new measurement techniques (DIC) and advanced numerical models, complex nonlinear effects (vaults, localised deformations, the influence of geometric backfill constraints) that were previously only hypothetical have been revealed and described.

Advanced photoelastic studies have enabled direct visualisation of the force chains and stress redistribution in granular assemblages [36,37]. However, existing studies typically analyse stress evolution independently of strain location and without explicit coupling to full-field displacement measurements. Although recent work has captured the dynamic behaviour of force chains [38,39], simultaneous measurement of both strain and stress fields under active ground pressure conditions remains unavailable.

However, they remain unrecognised, especially in terms of fully accounting for the deformation location and the soil-boundary behaviour. Nevertheless, the experience gathered has clearly brought the computational models closer to the actual behaviour observed in experiments. Although optical techniques are increasingly being used to observe force transfer at the molecular scale, their integration with numerical simulations remains under development. Recent advances in 3D imaging at the grain scale [40], optical force quantification [41], and techniques using transparent media for DEM calibration [42] demonstrate the importance of combining physical modelling with numerical methods. These developments clearly indicate the need for experimental benchmarks to validate and calibrate DEM simulations under controlled active-displacement conditions [43].

However, despite recent advances in DIC techniques and the increasing availability of granular photoelasticity, existing studies still analyse strain and stress fields independently, failing to provide a fully coupled comparison of both fields in real time during active wall motion. Furthermore, no previous experimental studies have provided synchronous full-field observations of strain localisation and stress redistribution based on tiny, incremental active motions, which limits current attempts to validate modern DEM and FEM predictions.

### Novelty and Contribution of the Study

The existing literature provides valuable information on active ground pressure and strain in granular materials. However, no previous work has captured a simultaneous full-field comparison of stress and strain evolution during active wall displacement, especially for tiny incremental displacements. Previous studies have focused on either displacement/strain fields [26] or stress redistribution [33] but not both simultaneously. This work introduces a novel integrated methodology enabling the synchronous recording of strain and stress fields in real time using a coupled DIC–photoelasticity system. This approach enables experimental observation of the early stages of micromechanical processes, identifies geometric correlations between stress chains and shear-strain locations, reveals the inverse stress–strain relationship, and can serve as a benchmark for calibrating DEM models and improving retaining-structure design. This study, therefore, provides a new experimental benchmark based on synchronous full-field observations of strain localisation and stress redistribution, supporting the development and validation of constitutive models and high-resolution numerical simulations of active ground pressure.

## 2. Materials and Methods

### 2.1. Digital Image Correlation for Displacement and Strain Fields

The development of high-resolution digital photography and appropriate software has enabled accurate analysis of deformation in granular media, based primarily on the digital image correlation (DIC) method. DIC was initially used in fluid mechanics [44] and later applied to displacement analysis in soil modelling [45,46].

In this study, displacement and strain fields in granular backfill were obtained using the digital image correlation (DIC) technique via the geoPIV algorithm developed by White and Take (geoPIV research group toolbox, University of Cambridge; http://www.geopivrg.com; accessed on 28 September 2025) [45]. GeoPIV is a subset-based cross-correlation method that tracks the rigid displacement of image fragments between successive digital images and is widely recognised in geotechnical engineering as an implementation of DIC.

All model tests were recorded with a standard Sony Cybershot digital camera (Sony Corporation, Tokyo, Japan) at 2560 × 1920 pixels in JPEG format. Several camera distances were tested, yielding an image scale of 9–13 pixels per millimetre; as a result, a single glass grain (d≈ 0.8−1.2 mm) was represented by approximately 50–150 pixels. The pixel-to-millimetre conversion factor was determined from the model’s known dimensions (wall height and field width) and verified at multiple points along the wall. For each stage of loading and wall displacement, a pair of images was acquired: one in polarised light (for photoelastic analysis) and one in non-polarised transmitted white light (for DIC analysis). Only non-polarised images were used to track displacements to avoid interference from local brightness changes caused by the photoelastic effect in individual grains.

In geoPIV, the non-polarised image is divided into square study windows, creating a regular grid. Following a convergence study similar to that described by Leśniewska and Muir Wood [47], several grid sizes (20 × 20, 30 × 30, 40 × 40, and 50 × 50 pixels) were tested, corresponding to average grain diameters of 2 × 2 to 5 × 5. The final analyses were performed using a 40 × 40 pixel search window, which provided a good compromise between spatial resolution and smoothness of the strain fields. The search radius in the second image was chosen to encompass the maximum expected displacement between two consecutive steps.

The geoPIV algorithm determines the displacement vector at the centre of each search window by maximising the normalised cross-correlation coefficient between the corresponding image subsets in two consecutive images. Sub-pixel accuracy is achieved by interpolating around the correlation peak, with a nominal displacement resolution of $10^{-2}–10^{-3}$ pixels [45]. The displacement fields were then numerically differentiated to obtain the components of the strain tensor, including the shear strain $\gamma_{xy}$ and the volumetric strain $\varepsilon_{v}$, which are discussed further in Section 3.

### 2.2. Photoelastic Studies of Granular Materials

Photoelasticity is an experimental method most often used to determine stresses in transparent, amorphous, and normally isotropic solid materials (e.g., glass, polycarbonate, synthetic resins) [48].

**Figure 1:**
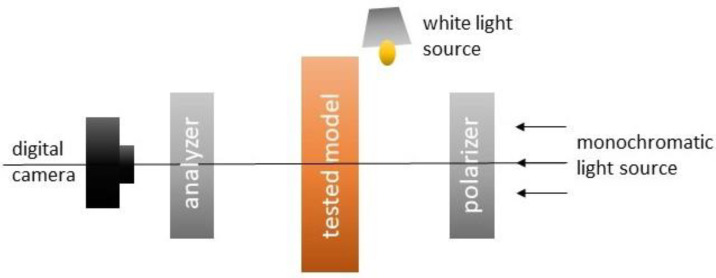
Optical setup for a transmission polariser.

Stress redistribution in a granular filling was studied using transmission photoelasticity. The model material consisted of glass grains (Starlitbeads1000) with a refractive index of n≈1.54, while the pores were filled with clove oil with a similar refractive index (n≈1.53), ensuring macroscopic transparency. Under stress, the glass grains exhibit stress-induced birefringence, generating characteristic interference patterns in polarised light. Experiments were conducted in a circular transmission polariscope equipped with a monochromatic sodium lamp, a polariser, two quarter-wave plates, and an analyser, arranged in a standard configuration for circularly polarised light (Figure 1). In this system, the transmitted light intensity I at a given point is related to the principal stress difference $\sigma_{1}\;-\;\sigma_{3}$ via the classical photoelastic relationships (1) and (2):

(1) $$I=I_{0}{sin}^{2}\Delta /2$$

(2) $$\Delta =[\frac{2C\pi d}{\lambda \left(\sigma_{1}-\sigma_{3}\right)}]$$

where I and $I_{0}$ denote the intensity of polarised light after passing through the model and the intensity of light entering the model, respectively, Δ denotes the difference in optical path lengths between the fast and slow rays [48], d is the model’s thickness, C is the optical elastochemistry constant of the material from which the model is composed, λ is the light wavelength, and $\sigma_{1}-\sigma_{3}$ is the difference in principal stresses at a given point in the model. As can be seen from Equation (2), photoelasticity is typically used to study flat models of constant thickness, which are placed in a polariscope and subjected to appropriate loads. In granular materials, this response manifests itself as bright force chains formed by highly stressed grains against a darker background.

In this work, the glass grain ensemble was thicker than a single grain layer, and the recorded images represent the depth-averaged photoelastic response of multiple grains along the optical path. For this reason, a fully quantitative inversion of the fringe patterns into absolute stress values at each point was not attempted. Instead, the analysis focused on the incremental changes in photoelastic intensity between successive loading stages, which provide information on the redistribution and localisation of contact forces within the granular framework. The processing procedure follows the approach proposed by Leśniewska and Muir Wood [47], which uses arithmetic operations on digital images to isolate changes in the photoelastic effect associated with small changes in wall displacement [47,49,50,51].

### 2.3. Integration of DIC and Elasto-Optic Methods

The most important feature of the adopted methodology is the integration of DIC-based displacement measurements with photoelastic stress visualisation within the same physical experiment. To ensure that both fields refer to the same set of grains, each experimental step was recorded twice: once under monochromatic, circularly polarised light (stress image) and once under white, unpolarized transmitted light (texture image for DIC). The circular polariscope’s optical system was therefore supplemented with an additional unpolarised light source and a digital camera, enabling the recording of images under both lighting conditions (Figure 1).

As discussed above, applying DIC directly to photoelastic images is not appropriate, because local brightness changes due to the photoelastic effect significantly alter the intensity pattern within each measurement window, leading to erroneous displacement estimates. By separating the stress (polarised) and strain (non-polarised) recordings, it is possible to obtain reliable displacement and strain fields from the natural texture of the glass–oil mixture while simultaneously recording the evolution of the force chains in the same region of the sample.

It should also be noted that for a transparent sample observed in transmitted light, both the DIC strain maps and the photoelastic images represent values averaged over the sample depth. The obtained strain and stress fields are therefore directly comparable based on the averaged depth. Further image processing, performed in MATLAB R2023a, included DIC analysis of the non-polarised images using geoPIV_RG, construction of incremental photoelastic response maps by subtracting successive polarised images, and superposition and geometric comparison of the shear and strain localisation zones with the evolution of the photoelastic force chains. This coupled optical methodology forms the core of the experimental approach presented in this work.

### 2.4. Experimental Setup for Model Tests

**Figure 2:**
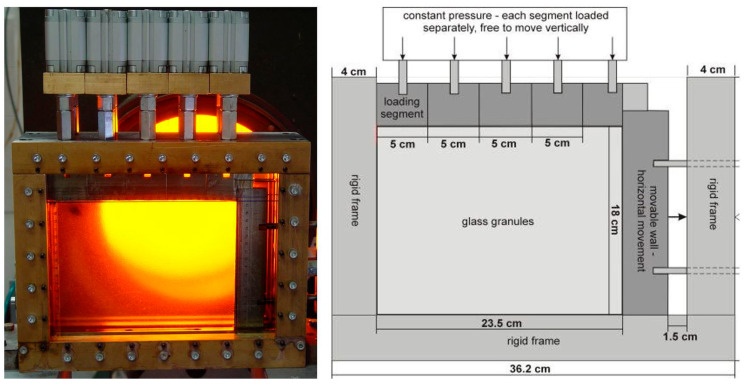
Test setup filled with glass granules under load. The black arrow in the diagram indicates the direction of wall movement.

The model tests were performed on a small scale in the test stand shown in Figure 2. It consisted of a metal box measuring 36.0 × 21.5 × 6 cm, in which two 2 cm thick panes of glass were embedded, a model of a vertical rigid wall, a loading system, and a system enabling horizontal displacement of the wall. The spacing between the panes, and therefore the model’s depth, was 2 cm. The dimensions of the stand were limited by the dimensions of the polariser and the analyser, which were components of the polariscope used in the tests (their diameter was 30 cm) [52,53].

The retaining wall, placed on the right side of the model (Figure 2), was connected to a calibrated screw outside the test stand via two horizontal guide rods.

During the test, by rotating the screw by a predetermined angle, the retaining-wall model gained freedom of movement under the pressure exerted by the granular material behind the wall and moved to the right. To induce a measurable elasto-optic effect in the tested material, the model had to be externally loaded. The external backfill load was applied using a system of five rigid metal blocks, each connected independently to a miniature hydraulic actuator to maintain constant pressure on the model surface (Figure 2). The hydraulic loading system allowed for pressures of up to 4 MPa. Next, in the photoelasticity studies, the model was filled from the bottom with immersion fluid, a loading head was applied, and the load program was first applied, followed by the wall displacement program. The basic displacement step was 1/20 of the screw pitch, which was 1.25 mm, corresponding to a unit wall displacement δd of 0.0625 mm or δd/H of approximately 0.035%, where δd denotes the unit wall displacement and H its height [52,53].

### 2.5. Experimental Materials

#### 2.5.1. Granular Materials

**Figure 3:**
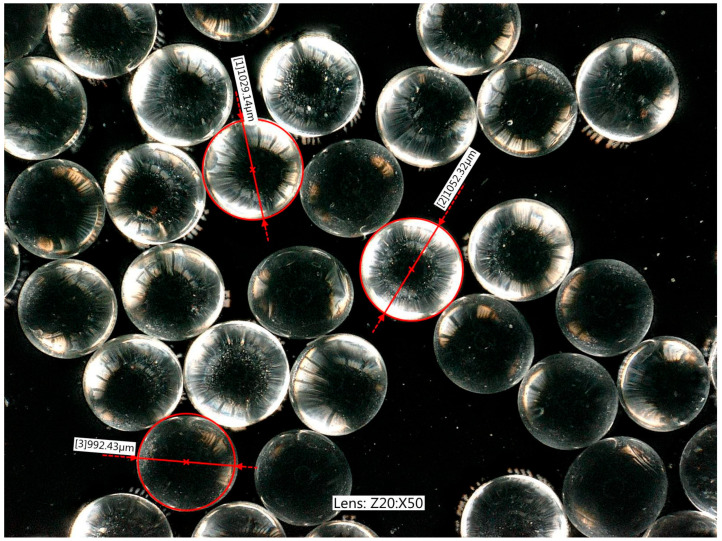
View of individual glass granules, magnified 50 times.

In most modelling studies, Starlitbeads1000 glass granules (Figure 3) with diameters ranging from 0.8 to 1.2 mm were used as the model soil. The internal friction-angle measured for them in the direct shear apparatus and the triaxial compression apparatus was in the range of $27^{{}^{\circ}}-34^{{}^{\circ}}$. They were made of sodium glass with a refractive index of 1.54. Other material parameters are given in Table 1.

#### 2.5.2. Immersion Liquid

In most of the modelling studies on glass granules, the pores of the medium were filled with an immersion liquid, namely clove oil, which has a refractive index of 1.53, close to that of the glass from which the glass granules were made.

#### 2.5.3. Model Test Procedure

A typical model test consisted of the following stages:

- Forming the sample by pouring;
- Filling the model with immersion fluid;
- Applying the load elements;
- Connecting the elements to the loading head, through which pressure is applied using a hydraulic pump;
- Loading the model (load range—0–4.0 MPa; minimum load increment of 0.2 MPa),
- Moving the wall by rotating the support screw;
- Recording the individual stages of the experiment with a digital camera, in normal and polarised light.

**Figure 4:**
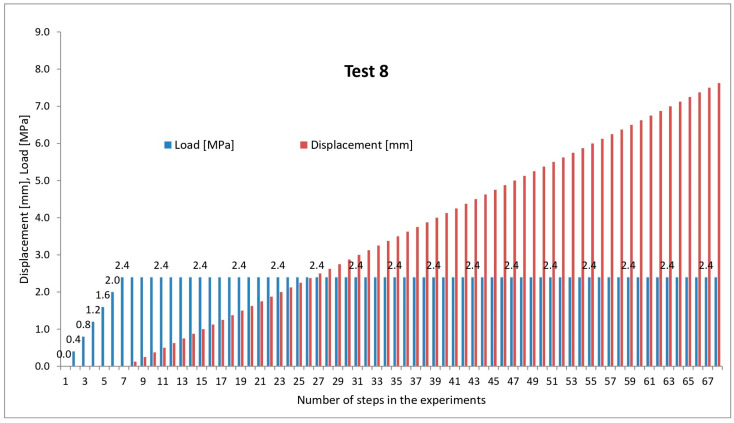
Representative model test procedure.

The course of each analysed test is presented as a bar graph (Figure 4), one showing the load level in MPa (blue bars) and the other showing the total wall displacement at a given stage of the experiment (red bars). Thus, the vertical axis represents both the load value and the cumulative wall displacement, while the horizontal axis represents the successive test steps, initially as the number of model-load increments and then as the number of wall-displacement increments. Figure 4 shows the typical course for most of the analysed tests: gradual application of an external load with the wall restrained until the assumed final load value is reached (maintained until the end of the test), followed by increments of wall displacement until the maximum assumed value is reached [52,53].

## 3. Results and Discussion

### 3.1. Experimental Results from the Model Tests

The direct results of the study were photographs of the model in polarised and non-polarised light, taken at each stage of loading and at each stage of displacement of the model retaining wall. The photographs were taken in .jpg format with a standard digital camera with a resolution of 2560 × 1920 pixels.

**Figure 5:**
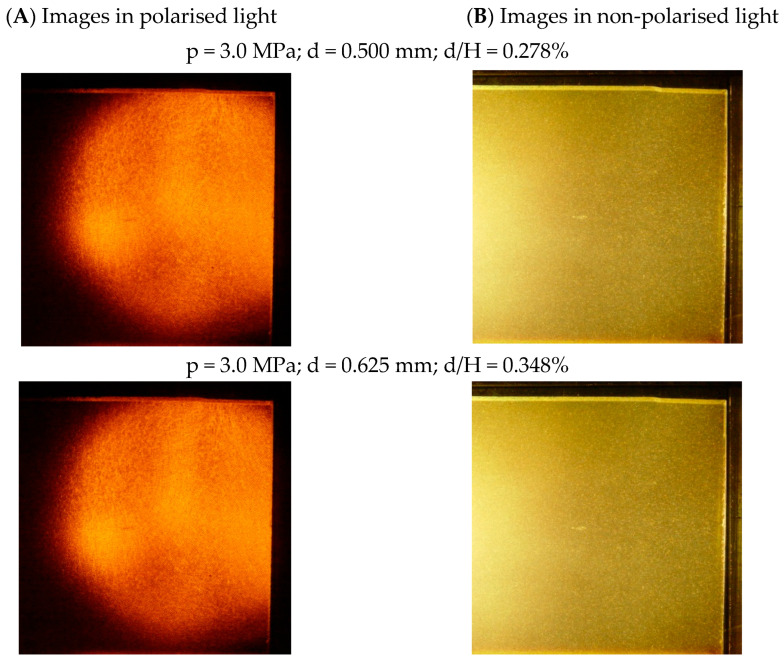
Example of original photos recorded at the beginning and end of the wall-displacement increment: (**A**) photos in polarised light; (**B**) photos in non-polarised light (p—load level [MPa]; d—total displacement [mm]; H—wall height [mm]).

Figure 5 shows two pairs of original photographs representing a single increment of wall displacement of 0.125 mm, obtained in polarised (Figure 5A) and non-polarised (Figure 5B) light. The circular shadows on the left side of the polarised light photographs indicate the boundary of the area illuminated by the sodium lamp in the polariscope. As shown in Figure 5, it is difficult to detect changes in the granular medium caused by a single displacement of the wall with the naked eye. Therefore, such photographs constitute raw materials that require further specialised analysis, especially in the case of photographs intended to determine the material’s deformation field, which are taken in non-polarised light.

**Figure 6:**
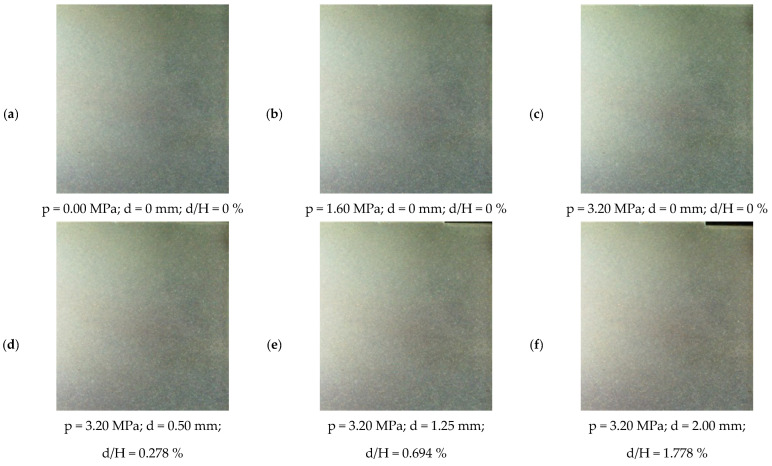
Fragment of Test 12, recorded in ordinary (unpolarised) light (p—load level [MPa]; d—total displacement [mm]; H—wall height [mm]); d/H—the ratio of the wall displacement to its height [%]; (**a**) p=0.00 MPa, d=0.00 mm, d/H = 0%; (**b**) p=1.60 MPa, d=0.00 mm, d/H = 0%; (**c**) p=3.20 MPa, d=0.00 mm, d/H = 0%; (**d**) p=3.20 MPa, d=0.50 mm, d/H = 0.278%; (**e**) p=3.20 MPa, d=1.25 mm, d/H = 0.694%; (**f**) p=3.20 MPa, d=2.00 mm, d/H = 1.778%) [56].

**Figure 7:**
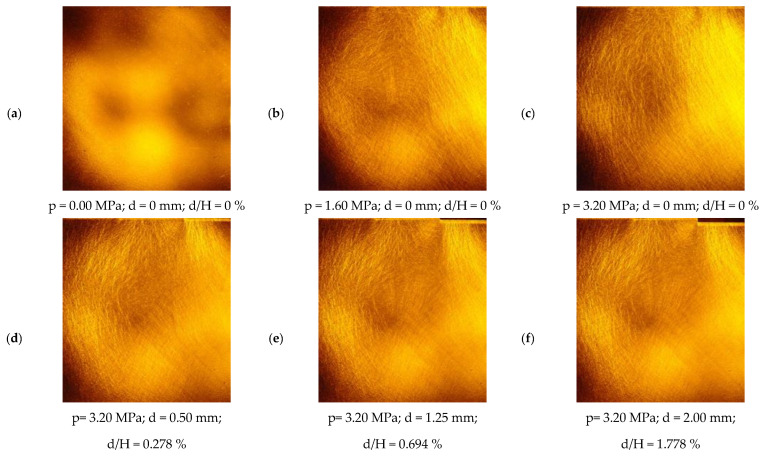
Fragment of Test 12 recorded in circularly polarised light (p—load level [MPa]; d—total displacement [mm]; H—wall height [mm]); d/H — the ratio of the wall displacement to its height [%]; (**a**) p=0.00 MPa, d=0.00 mm, d/H = 0%; (**b**) p=1.60 MPa, d=0.00 mm, d/H = 0%; (**c**) p=3.20 MPa, d=0.00 mm, d/H = 0%; (**d**) p=3.20 MPa, d=0.50 mm, d/H = 0.278%; (**e**) p=3.20 MPa, d=1.25 mm, d/H = 0.694%; (**f**) p=3.20 MPa, d=2.00 mm, d/H = 1.778%) [56].

The situation is somewhat different for images intended to record the force field (stress) in a granular medium—under certain conditions, they can be used directly, without special processing. This is illustrated in Figure 6 and Figure 7, which record six stages of a model test involving the retraction of a rigid wall away from the ground (to the right). Figure 6 contains images taken in ordinary (non-polarised) light, while Figure 7, corresponding to the same stages of the experiment, contains images taken in polarised light—this time, a distinct structure formed by “chains of loaded grains” is visible as the load increases. These chains, reminiscent of those obtained in the classic work of Wakabayashi and Drescher for crushed glass [54,55], are clearly visible when thermally tempered granules are used to construct the model. However, this results in a less distinct texture in images acquired under white (unpolarised) light (Figure 6), complicating subsequent deformation analyses using DIC. Chains of stressed grains in a medium composed of spheroidal glass granules were first observed by Muir Wood and Leśniewska [50].

Figure 7a shows that the glass panes constituting the front and back walls of the model contained residual stresses, which can be “removed” by treating this image as a background and subtracting it from subsequent images. The remaining photos, especially Figure 7b–f, show that changes in the arrangement of the chains of loaded grains, occurring under the influence of wall displacements, are, as before, difficult to see with the naked eye, so digital image processing is also necessary to isolate them.

**Figure 8:**
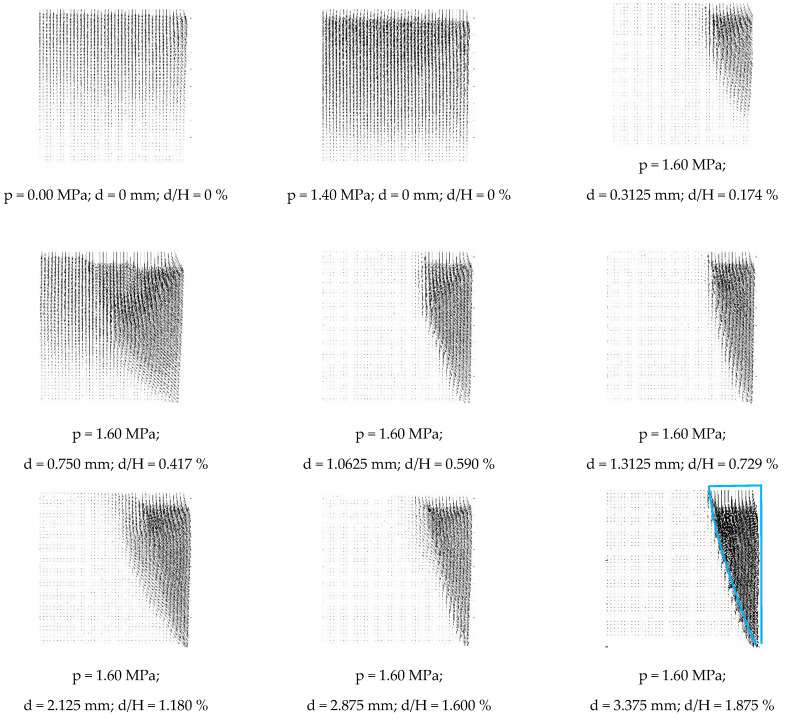
Displacement field for several selected steps of Test 4 (p—load level [MPa]; d—total displacement [mm]; H—wall height [mm]). The final picture includes an illustrative outline of the active failure wedge (blue line), which has been added to facilitate interpretation of the displacement field. All the remaining pictures are shown without annotations to preserve their original form.

From a methodological perspective, Figure 6, Figure 7 and Figure 8 illustrate the two complementary data streams that underpin this study. Images recorded in unpolarised light provide the texture necessary for DIC analysis and thus for constructing full-field displacement and strain maps, while images in circularly polarised light reveal the formation and reorganisation of photoelastic force chains within the granular framework. Although the individual displacement increments are very small (δd/H ≈ 0.035%), the synchronised acquisition of both image types at each loading and displacement stage enables a detailed reconstruction of the progressive development of the active damage mechanism, which is not possible in conventional model tests or in classical photoelastic studies of granular materials [54,55,57].

### 3.2. Results of Model Tests After Digital Processing

The fundamental effect of a properly performed DIC image analysis is the displacement field obtained by comparing two images corresponding to subsequent test stages (Figure 8 shows examples of the displacement field for selected steps of Test 4). It is important for the correctness of the DIC analysis that the difference in displacements corresponding to both compared images is not too significant—in the case of large displacements, image elements in the areas of cumulative deformation deform so much that they may not be correctly recognised by the image analysis program (in this case, geoPIV_RG). By knowing the displacement field, one can for example, calculate the deformation field, using the computational procedure in the finite element method, as described and implemented by White and Take in the geoPIV_RG program [46]. In the literature, for practical reasons, the most common searches are for shear-strain fields $\gamma_{xy}$ and volumetric strain fields $\varepsilon_{v}$, defined by relationships (3) and (4):

(3) $$\gamma_{xy}=\frac{\partial u_{x}}{\partial y}+\frac{\partial u_{y}}{\partial x},$$

(4) $$\varepsilon_{v}=\varepsilon_{x}+\varepsilon_{y}+\varepsilon_{z},$$

where $\varepsilon_{x}=\frac{\partial u_{x}}{\partial x}$, $\varepsilon_{y}=\frac{\partial u_{y}}{\partial y}$, and $\varepsilon_{z}=\frac{\partial u_{z}}{\partial z}$.

Before calculating the strains, however, it is important to remember that they must be determined from a physically meaningful displacement field. The condition for obtaining a correct displacement field is the proper division of the image into elements—their size cannot be too small (they will not be recognised due to insufficient visual information) or too large (they will be recognised, but they will cause excessive local averaging of the displacement field in areas of localised deformation). Therefore, the first step in deformation analysis using digital image correlation is to select (by trial and error) the fundamental dimension of the image division element.

To analyse the strain fields, the optimal size of the image subdivision element was determined not based on the displacement fields but on the shear and volumetric strain fields calculated from them. The criterion for selecting the element size was to achieve sufficient continuity of the strain field while maintaining an appropriate level of detail. The procedure for finding the subdivision mesh was analogous to the convergence study procedure in the finite element method. It involved performing a single-step analysis selected from three different model tests (Test 4) using four image subdivisions: a mesh composed of squares of 20×20 pixels (an element covering approximately 2×2 medium-sized grains), 30×30 pixels (~3 × 3 grains), 40×40 pixels (~4×4 grains), and 50×50 pixels (~5×5 grains). Regardless of the image element division used, in all cases, the location of the strain (both shear and volume) is clearly visible in bands of finite width. The arrangement of these bands is independent of the image division, but the image appears most “sharp” when divided into 40×40 pixel elements, and this division was used to analyse the modelling studies.

**Figure 9:**
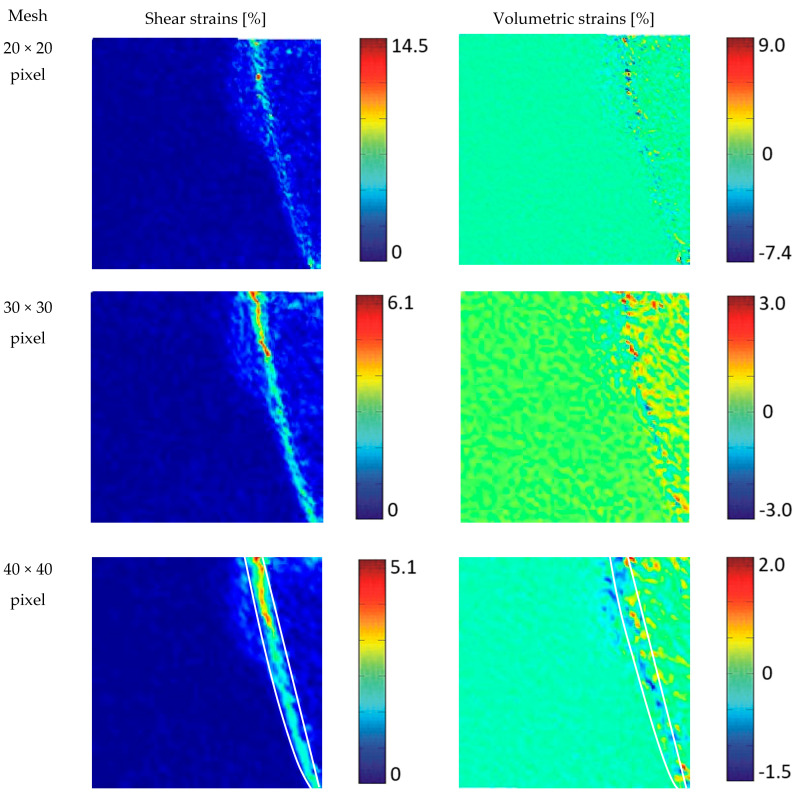

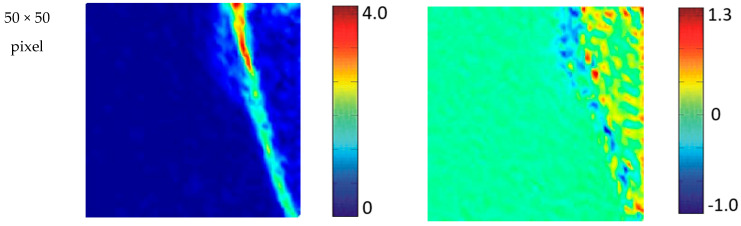
Grid division test based on photos 0173–0176 from Test 4. $\frac{d}{H}=1.6\%;$ p = 1.60 [MPa]; p—load level [MPa]; d—cumulative displacement [mm]; H—wall height [mm]. The white contour highlights the location of the shear band identified in both the shear-strain and volumetric-strain maps.

The displacement fields shown in Figure 8 and the corresponding shear and volumetric strain maps in Figure 9 clearly demonstrate the progressive nature of the active failure process. At very small wall displacements, localised shear zones nucleate near the bottom of the wall and gradually propagate towards the ground surface, forming a wedge-shaped region whose geometry is consistent with previous observations of shear band patterns in sands under plane-strain conditions [2,15,58]. The band inclination and its progressive widening with increasing displacement are also qualitatively consistent with the geoPIV observations of narrow backfills reported by Xu et al. [26], who showed that an active wedge develops through a sequence of localised slip surfaces rather than through instantaneous rigid body rotations. Compared with previous works, the present experiments capture these mechanisms at much smaller displacement increments, highlighting the ability of the adopted DIC procedure to determine the onset and early evolution of strain localisation under the influence of active ground pressure.

The shear displacements ($\gamma_{xy}$) and volumetric displacements ($\varepsilon_{v}$) in Figure 9 are presented as scaled colour maps. The magnitude of the shear strain varies from zero to the maximum value occurring in the analysed experimental step. The dark blue colour in the shear-strain maps indicates an area of minimal strain, while red indicates an area of maximum strain. Volumetric strain can have different signs: negative values indicate a volume increase (dilatation, stretching) while positive values indicate a volume decrease (compression), with the maximum stretch shown in dark blue and the maximum compression shown in red. In general, for a given experimental step, the maximum values of compression and tension are not equal [58].

### 3.3. Strain and Stress Fields

To achieve the research objectives, tests utilising the simplest wall-motion scheme—horizontal translation from the ground—were chosen because currently available full-field measurement methods (including image analysis methods) enable the recording of very small deformations that were previously considered immeasurable. Therefore, experiments were conducted with very small wall-displacement increments, amounting to fractions of a millimetre (δd/H ≈ 0.035% per step), which are significantly lower than those in most classical active earth-pressure tests [9,10,11,12,13,14,18,32,33].

The research methodology was based on the approach of Leśniewska and Muir Wood [47,49,50,51], combining photoelasticity with digital image correlation (DIC) to obtain, within the same experiment, independent yet synchronous information on the strain and stress fields. DIC-based analysis in unpolarised light provided full-field displacement and strain maps, while circularly polarised light images provided depth-averaged visualisation of the force-chain network in the granular framework. One key goal was to identify the geometric relationships between these two fields; i.e., to determine whether zones of intense shear or volumetric strain correspond to stress-concentration or stress-relaxation zones.

**Figure 10:**
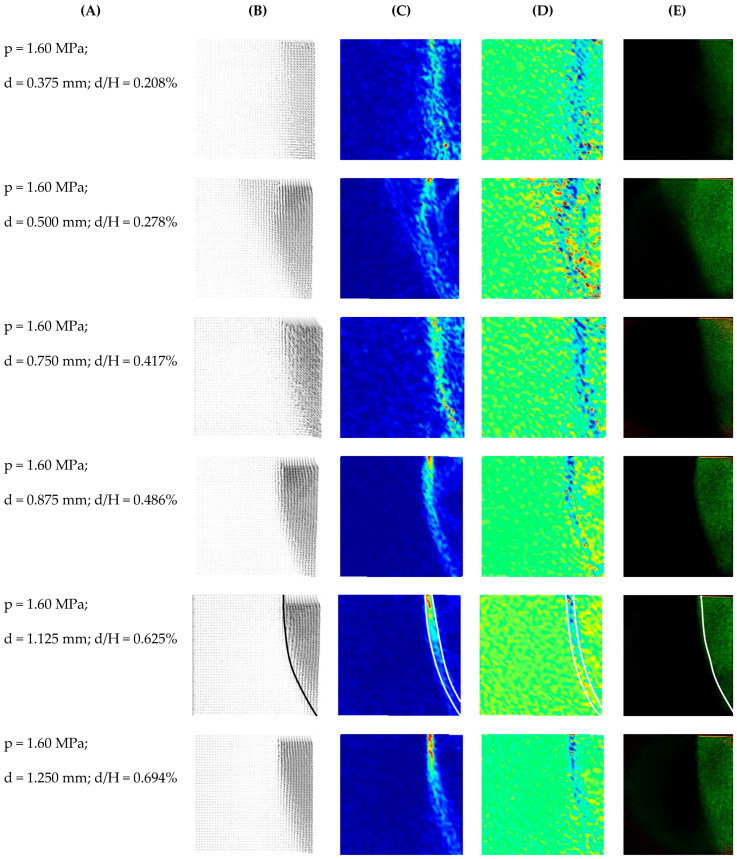
Comparison of displacement fields (**B**), shear strain (**C**), and volumetric strain (**D**) with changes in the stress field (E) for different stages of the selected test. (**A**) shows the image numbers. p—load level [MPa]; d—total displacement [mm]; H—wall height [mm]. For the representative step at d/H = 0.625%, additional contours were included to highlight key features of the deformation mechanism. In panels (**B**,**E**), the outlines of the active failure wedge are shown, while in panels (**C**,**D**) the contours indicate the location of the shear-strain localisation (shear band).

It is important to emphasise that the images in column E of Figure 10 are used solely to identify incremental changes in the depth-averaged photoelastic response between two successive loading or displacement steps. They are interpreted qualitatively as indicators of relative increases (brightening) or decreases (darkening) in stress rather than as fully calibrated three-dimensional stress fields.

Because the force-chain network is highly discrete (Figure 7), standard automated image processing techniques [59,60,61] could not meaningfully segment the photoelastic patterns. A fully quantitative inversion of fringe patterns into local stress values was beyond the scope of this work. However, by computing difference images between successive polarised photographs, it was possible to isolate incremental changes in photoelastic intensity for each displacement step. These differences could be visually compared with corresponding strain maps. Figure 10 shows such comparisons: columns B–D display the displacement and the resulting shear and volumetric strain distributions, while column E presents the incremental photoelastic response from differences between circularly polarised images.

A systematic comparison of columns C, D, and E in Figure 10 reveals a clear inverse relationship between strain localisation and stress redistribution. Areas of low shear and volumetric strain, such as the interior of the active wedge, coincide with brightened areas of increased photoelasticity in column E, indicating the presence of strong and relatively stable force chains. Conversely, zones of concentrated shear strain, where the material undergoes intense deformation, are associated with local stress relaxation, which is visible as darker areas in the difference images. If shear bands were hypothetically superimposed onto stress-change maps, they would lie primarily in the shaded (black/dark) regions, confirming that these bands correspond to paths along which the granular framework is unloaded [50,58,62].

Within this framework, Figure 10E is not intended to provide absolute stress magnitudes but to highlight the spatial correspondence between regions of stress redistribution and the shear-strain localisation patterns obtained from DIC.

This inverse stress–strain pattern is consistent with previous experimental and numerical observations of granular media, in which force chains carry most of the load and deform preferentially in the surrounding weaker regions [47,50,51,55,57,59]. However, previous photoelastic studies have either focused on highly idealised grain assemblies or have not provided a fully coupled comparison with full-field strain measurements. In contrast, the present results demonstrate, for the first time, a synchronous stepwise correlation between shear-strain localisation and the reorganisation of force chains in granular backfill under active soil pressure. This provides experimental support for the hypothesis formulated by Bransby and Milligan [12] that the reliable prediction of ground pressure requires simultaneous knowledge of the deformation field behind the wall.

Furthermore, the observed pattern of a relatively stiff low-strain wedge, confined by narrow shear bands and supported by strong force chains, offers a micromechanical explanation for the reduced active ground pressures reported under narrow burial conditions by Chen et al. [27], Xu et al. [26], and Bashir and Basha [33]. Their studies demonstrated that the effective active wedge contracts and is partially “bent” between the wall and the lateral boundaries, leading to lower net pressures than those predicted by classical ground pressure theories. The present experiments complement these findings by demonstrating how this bending is reflected in the internal arrangement of force chains and in the localisation of strains at small displacement increments. These conclusions may help calibrate advanced DEM and FEM models of active ground pressure [26,27,34,35,53], as well as for optimising retaining-wall design in cases where geometric constraints and strain localisation are important.

It should be emphasised that the formation of shear bands observed in this study corresponds well with theoretical descriptions of strain localisation in granular materials, where progressive damage is interpreted as a bifurcation phenomenon leading to non-uniform strain modes [63,64,65]. The existence of finite-width shear zones supports theoretical approaches based on non-local continuum or Cosserat models, which assume characteristic length scales and microrotation effects. This confirms that the experimentally observed patterns of strain and stress evolution are consistent with generally accepted theoretical models of localisation in granular media.

Furthermore, the inverse relationship observed between stress concentrations in the network of force chains and local shear and strain zones is consistent with the micromechanical interpretation of load transfer in granular media, where force chains carry most of the applied load, while strain develops in the surrounding, less-stressed regions [55,57,61]. This behaviour is also reproduced in numerical Cosserat models and nonlocal models, where stress redistribution accompanies strain localisation. Therefore, the present experimental results provide physical evidence in support of these theoretical formulations.

## 4. Limitations and Future Work

The optical methodology proposed in this work provides depth-averaged qualitative information on the redistribution of stress and strain in a quasi-2D ensemble of spherical glass beads. Several limitations should be highlighted. First, because the sample thickness exceeds that of a single-grain layer and the light path crosses multiple contact points, the recorded photoelastic response cannot be unambiguously converted into local, three-dimensional stress values; only relative changes in the integrated network of force chains are available. Second, DIC-based strain fields depend on finite image resolution and the chosen analysis window size, so kinematics at scales smaller than approximately 2–3 grain diameters remain unresolved. Third, the granular material and boundary conditions are idealised (glass beads, index-matched immersion fluid, transparent, smooth boundaries), which limits the direct extrapolation of results to field conditions and prevents the direct use of the current data as design curves.

Despite these limitations, synchronous stress and strain observations provide a useful mechanistic benchmark for calibrating and validating numerical models. Future work will therefore focus on combining the current experiments with DEM and FEM simulations, where the model’s contact forces or stress tensors are adjusted to reproduce both the measured boundary loads and the DIC-based strain maps. Such inverse or hybrid experimental–numerical approaches offer a promising avenue for a more objective quantification of internal stress fields, consistent with the observed evolution of shear bands and force chains. Furthermore, extending the methodology to other displacement modes and grain morphologies, as well as using advanced photoelastic force inversion or tomographic techniques, could further improve the quantitative interpretation of stress redistribution in granular media under active pressure conditions.

## 5. Conclusions

This paper presents a novel experimental methodology that combines digital image correlation and granular photoelasticity to obtain synchronous measurements of the full strain and stress field in granular backfill subjected to active soil pressure. The results show that at very small increments of wall displacement (δd/H ≈ 0.035%), shear localisation begins at the bottom of the wall and gradually develops into an active wedge through a sequence of narrow shear bands rather than through the instantaneous formation of a classical slip surface.

The most important result of this study is the experimental evidence of an inverse relationship between shear-strain localisation and stress redistribution: regions of intense shear coincide with local stress release, while regions of low strain are associated with persistent force chains. This inverse stress–strain interaction, observed step-by-step here for the first time, provides a micromechanical explanation for the reduced active earth pressures observed under geometric-constraint conditions and offers valuable benchmarks for calibrating numerical DEM models.

The integrated optical methodology used in this work builds on previous experimental approaches, enabling synchronous depth-averaged visualisation of both the strain and force-chain evolution during controlled active motion. Therefore, these results provide new insights into the micromechanics of active pressure, highlight the role of progressive strain localisation, and open up perspectives for improved retaining-wall designs in cases where small displacements and granular bulges can significantly modify earth-pressure distributions.

Future research will focus on numerically reproducing the observed stress–strain evolution and on extending the methodology to different displacement modes and grain morphologies to further develop and validate constitutive descriptions of granular materials under active pressure conditions.

## Figures and Tables

**Table 1:** Material properties of Starlitbeads1000 granules.

**Glass Granules**	**Median Grain Diameter** $d_{50}$ **(mm)**	**Uniformity** **Coefficient** U	**Volumetric Weight of the Soil Skeleton** $\gamma_{d}$ **(**$kN/m^{3}$**)**	**Relative Sensity** $I_{D}$
	1.1	1.1	16.80	0.80

## Data Availability

The original contributions presented in the study are included in the article; further inquiries can be directed to the corresponding author.
